# A Novel Approach for Simulation of Automotive Radar Sensors Designed for Systematic Support of Vehicle Development

**DOI:** 10.3390/s23063227

**Published:** 2023-03-17

**Authors:** Zoltan Ferenc Magosi, Arno Eichberger

**Affiliations:** Institute of Automotive Engineering, Graz University of Technology, Inffeldgasse 11, 8010 Graz, Austria

**Keywords:** automated driving, radar sensors, sensor modelling, virtual testing

## Abstract

Despite the progress in driving automation, the market introduction of higher-level automation has not yet been achieved. One of the main reasons for this is the effort in safety validation to prove functional safety to the customer. However, virtual testing may compromise this challenge, but the modelling of machine perception and proving its validity has not been solved completely. The present research focuses on a novel modelling approach for automotive radar sensors. Due to the complex high-frequency physics of radars, sensor models for vehicle development are challenging. The presented approach employs a semi-physical modelling approach based on experiments. The selected commercial automotive radar was applied in on-road tests where the ground truth was recorded with a precise measurement system installed in ego and target vehicles. High-frequency phenomena were observed and reproduced in the model on the one hand by using physically based equations such as antenna characteristics and the radar equation. On the other hand, high-frequency effects were statistically modelled using adequate error models derived from the measurements. The model was evaluated with performance metrics developed in previous works and compared to a commercial radar sensor model. Results show that, while keeping real-time performance necessary for X-in-the-loop applications, the model is able to achieve a remarkable fidelity as assessed by probability density functions of the radar point clouds and using the Jensen–Shannon divergence. The model delivers values of radar cross-section for the radar point clouds that correlate well with measurements comparable with the Euro NCAP Global Vehicle Target Validation process. The model outperforms a comparable commercial sensor model.

## 1. Introduction

Driving automation is intensively developed for reasons of road safety, driver comfort, energy efficiency, and traffic flow. It also introduces new mobility concepts, transforming the vehicle manufacturer (OEM) into a mobility provider. The need for virtual development for the safety validation of advanced driver assistance systems (ADASs), SAE Levels 0–2, and the higher-level automated driving functions (ADFs), SAE Levels 3–5, has come along with the drastic increase in the number of on-road test kilometres. Among others, Kalra et al. [1] quantified the total number of test kilometres required for safety validation. Depending on which statistical consideration is focused on and how the statistical questions are posed, they reported values ranging from 1.6 million to 11 billion km.

This has led to the integration of more and more vehicle subsystem or even full-vehicle models into the engineering process, which in turn has led to the systematic use of different simulation techniques through the whole vehicle development process. Virtual verification and validation (V&V) allows for early system concept proof and has become the state of the art in the automotive industry. In terms of driving automation, the key issue in virtual V&V is the implementation of machine perception. This requires virtual sensor models with sufficient prognosis quality. Although sensor models can be developed in many different ways, there are still some challenges to overcome. For example, the user should be able to easily simulate and parameterise machine perception models with a level of accuracy comparable to real driving or development requirements. In addition, for implementation in vehicle simulation, a high computing power is required for stable and robust simulation performance. Due to the above-mentioned need for virtual V&V, sensor models of different complexity and accuracy are required at different stages of vehicle development to fully satisfy the respective requirements. Therefore, in a previous work [2], we introduced a novel method for classifying radar sensor models found in the literature according to the stages of the vehicle development process. Based on the vehicle manufacturers’ requirements, we have also assigned them to these stages depending on their applicability. The radar sensor models considered were classified as follows:

- Operational Models (OMs): Generic sensor models can be easily and rapidly parameterised without knowledge of the specific perception sensor technology. Usually, the perception concept can be derived by focusing only on some typical geometric sensor properties such as field of view (FOV), detection range, etc.
- Functional Models (FMs): Stochastic, phenomenological, and data-driven modelling techniques are considered for subsequent investigations after the concept phase. In contrast to OMs, FMs require more detailed information about the sensor technology under consideration, but typically do not address the internal function of the HW/SW components of a real sensor. The functional representation of radar detection resulting in an object list can be modelled by the simulation of a simplified antenna pattern and the uncertainty of real sensors.
- Technical Models (TMs): Tailor-made sensor models for over-the-air (OTA) radar target stimulator test benches that support X-in-the-loop methods in the vehicle engineering process. A radar point target can be stimulated to validate basic sensor functionality such as bus communication. State-of-the-art OTA test benches require a reduced object list with position, distance, speed, and signal strength to generate a radar signature.
- Individual Models (IMs): Physics-based models for verification of sensor components and perception algorithms. Technology- and HW-specific parameters as well as detailed technical information of sensors are required for qualitative performance analysis. IMs are the most accurate models at the cost of high computational effort and real-time capability. Reliable modelling is only possible with the expertise of sensor suppliers.

The overall scope of this research project was the development and implementation of a complete process chain with the aim of developing a scalable radar sensor model that can be classified into operational, functional, or technical model for use throughout the vehicle development process. In order to fully meet the requirements, a three phase perception sensor modelling framework has been proposed.

The radar sensor model presented here uses physical modelling approaches where possible, or otherwise, mathematical approximations leading to a semi-physical modelling technique. The identification of the phenomena required for the synthesis was based on observations of real sensor recordings taken during the execution of specific driving manoeuvres. The classification of the identified phenomena was based on radar theory without external high-frequency measurements and without looking into the internal HW/SW architecture of the real sensor, see Section 3.2. The proposed modelling approach gives similar results to the real sensor and the main contributions of the research are summarised below:

- To the authors’ knowledge, this is the first time that the asynchronous output data streams of two automotive radar sensors of the same type, but with different configurations in terms of output processing level, have been recorded synchronously and analysed by projecting them onto each other in order to identify sensor-specific phenomena.
- The modelling approach is semi-physical by incorporating the characteristics of the directional antenna, the propagation factor, and some backscattering properties into the radar equation. In addition, physical effects such as Doppler and µDoppler, derived from measurements with the real radar sensors, have also been incorporated. By using these effects, a much more realistic radial velocity simulation can be achieved. The proposed model synthesises the radar point cloud and radar cross-section (RCS) taking into account the subsequent detection algorithms.
- As the required input from the sensor system supplier is limited to public information from datasheets and access to the radar point cloud, an extensive driving scenario catalogue was defined and performed to derive critical sensor characteristics and parameters. An off-line analysis tool was then developed to synchronously overlay ground truth information and all asynchronous sensor outputs.
- The model is, in real time, capable and ready for implementation on different X-in-the-loop test benches, for example, for over-the-air radar simulation test benches.
- The model is intentionally prepared to be used over the overall development process ranging from the concept phase to future virtual vehicle homologation.

The remainder of this paper is structured as follows: Section 2 reviews existing representative works from the perspective of the system integrator. Section 3 describes in detail the mathematical and physical approach of the sensor model. Section 4 presents the fidelity of the model in comparison with experimental data and Section 5 compares the performance to a commercial radar sensor model and summarises the findings.

## 2. Related Work

Sensor models are intended to reproduce measurement uncertainty, physical characteristics of the real sensor, and phenomena that may be associated with specific sensor technologies. In contrast to the real world, the virtual environment must also be modelled with a sufficient level of detail, resolution, and accuracy, taking into account the characteristics of the sensor being modelled. Concerning virtual environments, the authors in [3] divide the state-of-the-art environment simulation methods into two general classes regarding modelling complexity. One class includes object list-based environment modelling, relying on ground truth provided by simulation software and providing perfect detection for sensor models. The other class includes all modelling methods that can be used to generate detailed or even realistic synthetic environment data in the form of low-level sensor data. In addition to this, the interface or the channel between the virtual environment and the sensor model also has to be modelled in an appropriate way [4].

To support X-in-the-loop testing methods during the vehicle development process, a wide range of commercial or open-source simulation software is available to the automotive industry. Referring to our previous work [2], some examples are given: in [5]: TASS-PreScan, dSpace-ASM, in [3]: TESIS Dyna4-Driver Assistance, MathWorks-ADAS Toolbox, in [6]: CARLA, AirSim, DeepDrive, Udacity, or in [7]: CarMaker from IPG Automotive GmbH., VIRES-VTD. These software packages provide a variety of interfaces for modelling perception sensors at different levels of complexity, but their parallel use is often limited, whereas in real application data, the fusion of multiple sensors is state of the art.

They are only available separately for each use case, as only one interface is available at a time.

In line with our terminology for classifying sensor models, OMs offer considerable efficiency at the design stage to validate the perception concept. These simplified generic, ideal, ground truth, or geometric models simulate perfect sensor behaviour that accurately detects all objects in the idealised sensor FOV. Parameterisation is simple as the user only needs to configure the required sensor coverage area by defining the geometric outline of the sensor’s FOV. Since most of these models, which can be classified as OMs, are provided with the above-mentioned simulation software, they form a stable and efficient basis for further feature integration. The authors in [8] propose a generic modular design for modelling perception sensors based on virtual objects provided by a simulation framework. The work in [9,10] presents implementations based on a modular design. In the first step, the geometrical characteristics of the sensor and the environment are taken into account in order to calculate the reference point and the occlusion decision. Scene modelling, regardless of sensor type, allows for the rapid creation of system/environment relationships and accelerates virtual concept validation.

In contrast to the above-mentioned low computational cost and simplicity in simulation, IMs collect sophisticated perception sensor models with a physics-based modelling approach. IMs make it possible, if the technology used and the specific parameters of the hardware and software are known, to reproduce real sensor characteristics even without abstracting the environment. Since all the technological knowledge is available at the sensor supplier’s site, simulations of any sensor part down to the semiconductor components can be carried out. Examples include ray tracing (RT) and any time-domain electromagnetic wave simulation-based modelling techniques can be found in [11,12,13,14,15,16,17,18,19]. The advantages of ray tracing is that each path taken by a virtual ray can be computed individually, resulting in a high-fidelity simulation, but at the cost of increased hardware requirements. In the literature, the use of the NVIDIA^®^ OptiX™ ray-tracing engine on NVIDIA GPUs dominates when considering the use of ray-tracing techniques for mm-Wave radar simulation [20].

FMs offer the widest applicability when considering the phases of vehicle development. The modelling design can vary over a wide range to accommodate different design considerations driven by the right trade-off between complexity, fidelity, and computational cost. In these cases, for example, the real material description model can be replaced by a probabilistic material model as proposed in [12], or the radiation pattern of the antenna can be assumed to be known [19], when complex objects are replaced by multiple scatterers [13], or when all metallic surfaces are treated as perfect conducting (PECs) in [16], whereas all other materials are considered to be absorbers in [21]. Unlike OMs, FMs contain more information and detail about real sensor characteristics and may require moderate sensor knowledge. The modelling task of an FM typically deals less with the internal signal and data processes of the real sensor and focuses on reproducing the effects that distinguish the sensor output from the reference data. Several solutions to represent different sensing tasks can be found in the literature. Stochastic [22], phenomenological [10], data-driven [23], and semi-physical [24] modelling methods are the most commonly used. The authors in [25,26] show that with a non-parametric modelling approach, sensor detection range, occlusions, latencies, ghost objects, and object loss can be modelled in a realistic way without explicit programming and can be simulated efficiently in real time. The same concept, where the geometric information of the target is transformed into the sensor model and then signal noise and statistically based signal loss are superimposed on the original signal, has been developed in [10,27]. In order to obtain a statistical distribution that can be applied by the model, a large number of experiments are required. Furthermore, in the real world, there are often critical parameters that influence the perception results, so a given statistical distribution does not necessarily correspond to the perception performance of the detector. Therefore, data-driven approaches based on machine learning (ML) and deep learning (DL) were introduced. In general, ML and DL-based approaches have improved significantly over the past decade, driven by the availability of advanced GPUs accelerated by its highly parallel architectures. The application of ML-based perception algorithms in optical systems for object recognition and classification is already being used in commercial systems [28] and its feasibility to interpret radar returns was demonstrated in [29] and for target classification in [30,31]. Furthermore, the authors in [32] proposed an ML-based method for the perception of airborne radar and compared its performance with that of a DL algorithm based on recurrent neural networks (RNNs). DL is now being applied to a wide range of other areas, including speech recognition, image search services on big data, medical diagnosis [33], and much more challenging tasks. In the areas of healthcare and surveillance, the authors of [34] proposed a pattern-balanced semi-supervised DL model for imbalanced human activity recognition (HAR) on a multimodal dataset. In addition, the authors in [35] introduce two DL-based frameworks for electroencephalography-based (EEG) human intention recognition applied to a brain–computer interface (BCI). In the automotive domain, using the dynamic encoder–decoder modelling framework, an EEG signal-based driver drowsiness estimation application was introduced in [36]. Coming back to simulating perception sensors, a radar data simulation using deep generative networks was presented by the authors in [37]. For further reading, ref. [28] provides a good overview of the many applications of ML and DL algorithms in the context of automated driving (AD).

Unlike the broad applicability and availability of OMs and FMs, TMs collect sensor models that provide a customised object list representing a radar signature used as input to OTA radar target stimulators. This allows for the radar signature to be generated by the high-frequency transceiver in the form of a point target, and requires much less computing power than its simulation-only counterparts. A typical radar signature for X-in-the-loop applications consists of the frequency shift $f_{d}$ due to the Doppler effect proportional to the radial velocity, the propagation delay δt proportional to radial distance, the angular directions (azimuth Φ, elevation Θ), and the RCS σ, which describes the effective area of the objects to be stimulated. Despite the focused application and the reduced object list, a number of different modelling approaches can be found in the literature for the generation of realistic radar signatures. A phenomenological approach can be found in [10,26,38], and a simulation of object list based on multiple virtual scattering centres was proposed in [39]. More advanced models apply a physical approach to the representation of objects. The authors in [16] introduce a ray-tracing-based OTA tool-chain using a bi-directional reflectance distribution function (BRDF) method.

Summarising the above, it is evident that sensor models of varying complexity and fidelity are in demand and have been developed accordingly. Although commercial software tools provide a variety of generic perception sensor models, they are also only available separately for each use case. This inevitably leads to the inefficient use of different modelling approaches with different, sometimes unknown, parameter sets throughout the vehicle development process.

To address this problem, a modular radar sensor model based on a semi-physical modelling approach is presented. By means of the provided interfaces between the sequentially connected modules, the complexity and the fidelity can be varied, allowing for the classification of the sensor model as OM, TM, or FM.

## 3. Model Development Procedure

The following main considerations were used to develop the modelling methodology:

- Physical modeling where possible, otherwise mathematical approximation based on experimental data.
- Systematic modular structure in which the modules are connected via defined interfaces.
- Sufficient fidelity to reality or to the respective vehicle development phase to support the safety validation. Component testing that is the responsibility of system and component suppliers is not addressed.
- Implementation in commercial ADAS testing software.
- Real-time performance for X-in-the-loop testing.

**Figure 1:**
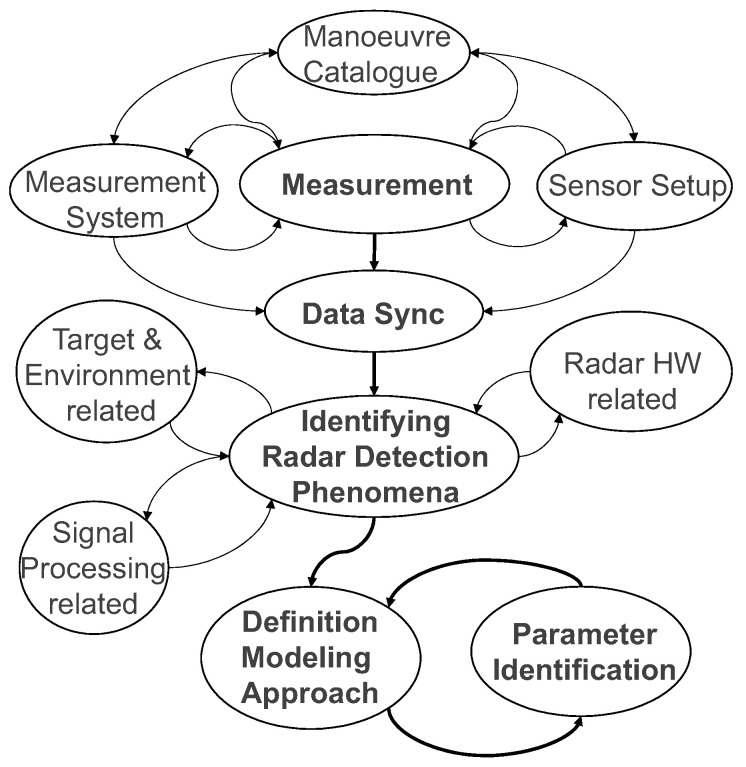
Process flow to derive the radar sensor model.

Accordingly, as shown in Figure 1, the first phase is to define and implement the measurement system and the test vehicles, followed by the definition of driving scenarios to challenge the sensors. The specific arrangement of the two automotive radar sensors, equipped with an open data interface, was also defined here to overcome the lack of confidential information through measurements and to allow for the observation of phenomena. In the second phase, the observed phenomena related to targets and environment, sensor hardware, or signal processing were identified and classified, from which the generic modular structure of the presented model was derived. Finally, in order to allow for a quantitative comparison of the simulation results, an evaluation method was proposed in the third phase.

In the following, the modelling method of the radar sensor is presented in more detail.

### 3.1. Development of a Suitable Measurement Setup

The measurement setup developed allows for simultaneous measurements with two radar sensors of the same type. The sensors are configured differently with respect to the processing level of the output data. In order to generate a real sensor data stream, the sensor setup in this work consisted of two ARS-308 radar sensors from Continental Engineering GmbH. The ARS-308 radar sensor operates in the 77 GHz frequency band and applies Frequency Modulated Continuous Wave (FMCW) technology, modulated by fast chirped sequences [40]. In this modulation method, the carrier signal is modulated with a sawtooth waveform whose frequency varies linearly with time. The mechanically scanning antenna provides two independent scans in the range of 0.25–200 m for long-range detection and 0.25–60 m for short-range detection [41].

The radar sensor is configurable between two operating modes according to the processing level of the output data. Based on the terminology defined by Continental in the sensor datasheet, the first sensor was configured to output radar target information, which is updated in each scan period, and the second sensor was configured to output so-called radar object information, which is the output of an advanced perception and tracking algorithm. In the target mode, which was used for the experiments presented here, twelve parameters are available per target, the most important of which are range, azimuthal angle, speed, and RCS. The measurement system used allows for data to be timestamped and synchronised not only between the target and the ego vehicle, but also between the sensors and the vehicle CAN bus data streams using multi-phase sync clock technology. A detailed description of the measurement setup can be found in our previous work [42]. Subsequently, using a self-developed visualisation tool, we were able to analyse the behaviour of the real sensors in different driving scenarios defined in the manoeuvre catalogue.

### 3.2. Identifying Radar Perception-Related Phenomena

Based on our observations in accordance with our previous research [18], radar detection phenomena that can be related to some specific characteristics of the target and environmental elements (i–iii), the signal processing applied to the FMCW radar technology (iv–v), and the radar hardware (vi) are required to be implemented in the radar sensor model.

i Radar detections can be assigned to distinct areas within the gate window.
ii The characteristic fluctuation pattern of the measured RCS value [43].
iii Detection of occluded targets.
iv The micro-Doppler effect [44] on rotating wheels.
v The effect of a rapid change in the relative acceleration (jerk).
vi The sensor’s FOV, resolution, and separability as specified in the data-sheet [45].

Those detected phenomena led to the definition of the modelling approach and the related parameter identification as described in the next section.

### 3.3. Radar Sensor Model

**Figure 2:**
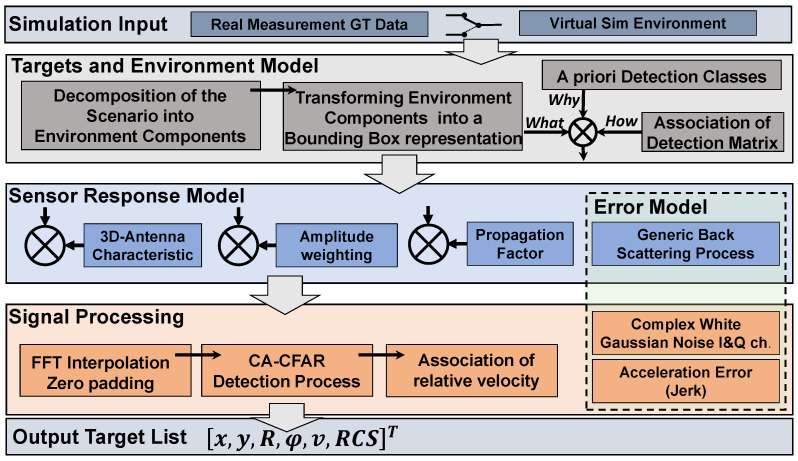
Modular structure of the radar sensor model.

With respect to our modelling objectives, the radar sensor model simulates detections represented in a 2D range-azimuth (RA) map, synthesising a square-law detector-like output. Accordingly, the distribution of the power of the back-scattered signal is assumed to have an exponential shape for each resolution bin. Furthermore, the basic radar equation was applied to the synthetic radar data to define the radar link budget [46] (p. 102) since the only information from a real radar sensor relating to the received signal strength is the radar cross-section expressed in ${\mathrm{dBm}}^{2}$. The effect of obstruction by preceding traffic objects is incorporated by calculating the local signal attenuation, which is a function of the radial distance to the radar and the 3D dimension of the shadowing object. The two-way propagation channel is characterised by small- and large-scale fading to represent the effects of multi-path wave propagation combined with range and medium-dependent free space signal attenuation. The modular structure of the sensor model shown in Figure 2 is described below.

#### 3.3.1. Simulation Input

The model development was based on real measurements, which were prepared for scenario replay in a digital twin for virtual testing. Thus, simulation input can be derived from both real measurements and virtual driving situations.

#### 3.3.2. Targets and Environment Model

**Figure 3:**
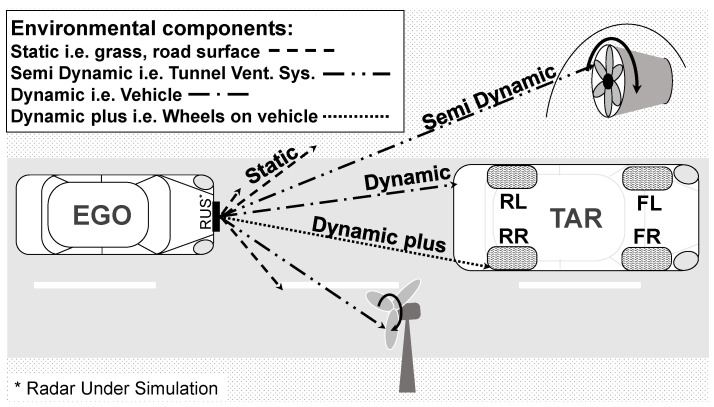
Environment classes for the sensor model.

This modelling step begins by decomposing the scenario into Environment Components (ECs) by assigning it to the Environment Classes shown in Figure 3. These are defined as static (road surface), semi-dynamic (tunnel ventilation systems), dynamic (vehicle body), and dynamic-plus (wheel on vehicle body) environment components. Despite the projection of the real sensor FOV into a 2D representation, we assume that the scenario is measured in all three dimensions. The virtual sensor FOV is then extended in elevation direction by the same number of receiver channels as defined in the real radar’s data-sheet for the azimuth direction. The scenario from a real measurement or from a virtual test drive are transformed into the 3D virtual sensor FOV with reduced resolution in all three dimensions. At this processing stage, the virtual sensor FOV is divided into 17 × 17 × 450 space bins (SBs) labelled with the space bin indicator (SBI), which corresponds to an angular resolution in both azimuth and elevation direction of 1.0625° and 0.45 m in range direction. This low-resolution spatial representation makes it possible to transform the environment components into a bounding-box representation.

In order to generalise the virtual radar detection behaviour, the following a priori detection classes (DCs) were defined for different radar detection modalities. Simple detection for scatterer in line of sight (LOS), scattered detection scatterer out of LOS (i.e., under body reflections), Doppler detection scatterer with relative speed difference to sensor and μDoppler detections scatterer with rotatory movement relative to its own body.

To implement the virtual detection modalities, a detection matrix combining the ECs and the DCs was defined, taking into account the FMCW principle and its powerful frequency analysis capability.

This assignment makes it possible to realise detections based on either the signal amplitude level or the relative velocity or in combination of both signals, as in the case of the real sensor. At the same time, this method allows for the model to be extended to include the separability function, which also uses the signal amplitude and/or velocity signals.

#### 3.3.3. Sensor Response Model

The sensor response model shown in Figure 2 represents, in a simplified form, the processes incorporating the observed and inferred effects that may be part of the RF front end of a real radar sensor. The output of this module is a superposition of all the effects that could have an impact on the strength of the received signal. The proposed modelling approach is based on the signal processing theory of linear time invariant (LTI) systems. To represent an LTI system, the impulse response function is used, which is also the output of the system when the input is a pulse. If the impulse response function of an LTI system is known, then the output can be calculated deterministically for any input. The impulse response of the proposed radar sensor model is the sum of the signals backscattered by each scatterer in a resolution cell of the sensor’s field of view. Accordingly, the statistical superposition of multiple scattering objects characterises the impulse response function of the system. In order to derive the impulse response function, the transition from the time domain to the spatial domain has been applied, an approach introduced by [47]. This transition means that frequency-dependent quantities are not represented as a function of time, but as a function of location. This is achieved by converting the time axis to the spatial axis via the speed of light.

In automotive radar applications, the EM wave propagation can be characterised by the fundamental effects of reflection and scattering. The EM wave is reflected when the irradiated object has a dimension much larger than the wavelength, while scattering occurs on large rough surfaces consisting of irregularities that have a small dimension compared to the wavelength. Accordingly, the backscattered signal in a radar image is composed of many small scattered emitters whose position is distributed across the field of view. As defined in Section 3.3.2, the sensor’s field of view is divided into spatial areas that represent the resolution cells. The resolution cell is then the smallest area in which the contribution of (finitely) many, interfering scatterers is combined into one amplitude value. According to [48], the backscattering process in one resolution cell can be defined by calculating the sum of the contribution of the particular scatterer located at *x*,*y* in the sensor‘s coordinate system. The reflection coefficient $\sigma_{\mathrm{SBI}}$ for every resolution cell can then be written as

(1) $$\sigma_{\mathrm{SBI}}=Pois_{\mathrm{SBI}}\cdot \sum_{i=1}^{N}b_{i}\delta \left(x-x_{i}\right)\delta \left(y-y_{i}\right)\;,$$

where $b_{i}$ is a random number, and δ is the impulse of the scatterer. Since FMCW signal processing is characterised by the coherent process interval (CPI) in terms of the number of the integrated phase-locked chirps required for a given velocity resolution $N_{CPI_{Tx}}$, we extend Equation (1) by the random variable $Pois_{SBI}\left(r,\alpha \right)$,

(2) $$Pois_{\mathrm{SBI}}\left(r,\alpha \right)=\frac{\alpha^{r}}{r!}\cdot e^{-\alpha \;}\;,$$

with

(3) $$\alpha =N_{{\mathrm{CPI}}_{Tx}}/2\;.$$

We define the sum of the individual scatterers for each *N* and $b_{i}$ as 1 and assume that the backscattering process is a random sequence within a CPI and can be described as a Poisson distribution. With this simplification, the backscattered signal amplitude is a function of a statistical random Poisson process representing the number of coherently received chirps from every resolution cell labelled with the SBI; see the section on targets.

Considering a typical mounting geometry for a motor vehicle radar sensor, and assuming that the sensor emits only in the horizontal polarisation plane, the impulse response $g_{s}$ for a point scatterer *s* located at a distance $r_{s}$ at any angular position $\vartheta_{s}$ for elevation and $\phi_{s}$ for azimuth can be defined as

(4) $$g_{s}\left(r_{s},\vartheta_{s},\phi_{s}\right)=D\left(r_{s},\vartheta_{s},\phi_{s}\right)\cdot R\left(r_{s},\vartheta_{s},\phi_{s}\right)\cdot F\left(r_{s},\vartheta_{s},\phi_{s}\right)\cdot e^{-i\cdot 2k_{\lambda }\cdot r_{s}},$$

where $D(r_{s},\vartheta_{s},\phi_{s})$ represents the effect of the directional characteristic of the antenna also considering the sidelobe reflections, $R(r_{s},\vartheta_{s},\phi_{s})$ represents the amplitude response (amplitude decay/path loss) as a function of radial distance to the scatterer, $F(r_{s},\vartheta_{s},\phi_{s})$ represents the impact of propagation effects, and $e^{-i\cdot 2k_{\lambda }\cdot r_{s}}$ the phase term of the back-scattered signal with $k_{\lambda }$ as the wave number.

According to the authors in [47], the radar equation can be determined from the quotient of the transmitted and received power $\frac{P_{Rx}}{P_{Tx}}$, by forming the absolute square of the impulse response function with regard to power $g_{sP}\left(r_{s},\vartheta_{s},\phi_{s}\right)$,

(5) $$|g_{sP}\left(r_{s},\vartheta_{s},\phi_{s}\right){|}^{2}=\frac{P_{Rx}}{P_{Tx}}=\frac{\lambda_{c}^{2}\cdot G_{Tx}\left(\vartheta_{s},\phi_{s}\right)\cdot G_{Rx}\left(\vartheta_{s},\phi_{s}\right)\cdot \sigma_{s}}{{\left(4\pi \right)}^{3}\cdot r_{s}^{4}}\cdot F{\left(r_{s},\vartheta_{s},\phi_{s}\right)}^{4},$$

where $\lambda_{c}$ is wavelength of the carrier frequency, $G_{Tx}$ and $G_{Rx}$ the gain of transmitter and receiver antenna, respectively, and $\sigma_{s}$ is the reflection coefficient of the point scatterer.

As we are interested in expressing the amplitude *A* of the impulse response $g_{s}\left(r_{s},\vartheta_{s},\phi_{s}\right)$ in terms of the transmitted power, (5) can be rewritten according to [49] into

(6) $$g_{s}\left(r_{s},\vartheta_{s},\phi_{s}\right)=\frac{1}{4\pi r_{s}^{2}}\sqrt{\frac{2P_{Tx}\cdot G_{Tx}\left(\vartheta_{s},\phi_{s}\right)\cdot \sigma_{s}}{\varepsilon_{0}\cdot c}}\cdot e^{-i\cdot 2k_{\lambda }\cdot r_{s}}\cdot |1+\Gamma \cdot e^{i\cdot k_{\lambda }\Delta r_{s}}{|}^{2},$$

where $\varepsilon_{0}$ is the permittivity of the vacuum, $k_{\lambda }$ is the wavenumber, *c* the speed of light, and $\Gamma \cdot e^{i\cdot k_{\lambda }\Delta r_{s}}$ is the Fresnel reflection coefficient.

- Amplitude weighting as a function of distance. In radar theory, the power of the received signal is expected to be proportional to the fourth power of the distance to the scatterer or target. Due to the many simplifications applied for the simulation, this rule does not fit our radar link budget [46] (p. 102) compared to the real sensor output, so we introduced a new amplitude weighting function in the form of an exponential decay. The new exponential amplitude decay $R(r_{s},\vartheta_{s},\phi_{s})$ is still the function of the range and can be expressed by definition as follows:
  (7) $$R\left(r_{s},\vartheta_{s},\phi_{s}\right)=R_{0}e^{-\tau \cdot r_{s}},$$
  where $R_{0}$ is the minimum detection range of the radar under simulation (RUS), τ is the rate constant and if it is less than zero, it represents a decay.
- 3D Antenna Characteristic The antenna is the coupler that transforms the EM waves of the propagating channel into current for the RF electronic components in receive mode, and vice versa in transmit mode of the radar sensor. Radar antennas are characterised by their directivity, which can be described by the antenna pattern. The ARS-308 industrial radar sensor is designed with a unique mechanically scanning antenna concept, which is a improvement of the folded parabolic antenna [50]. A prototype of a high-resolution imaging radar sensor for automotive applications with a similar narrow beam antenna concept was presented by the authors in [41]. They achieved a half-power (3 dB) beam-width of 1.6 degrees in azimuth and 4.2 degrees in elevation. According to [51] (p. 279), in order to achieve an operational antenna performance with an asymmetrical beam width and a low sidelobe level, different types of aperture antennas can be considered. In particular, a good result can be obtained for two-dimensional planar arrays in the form of a rectangular radiating surface [52] (p. 316) with cosine-weighted aperture irradiances. Certain aperture distributions (e.g., Hamming or Taylor) have a lower first sidelobe, but cosine shaping is appropriate for the modelling approach introduced here [51] (p. 232). Accordingly, the 1-D normalised antenna pattern for the cosine aperture distribution over one angular direction or plane of a rectangular aperture is calculated as follows:
  (8) $$E\left(x_{a},\phi_{s},\lambda_{c}\right)=\frac{\pi }{4}\left[\mathrm{si}\left(\Psi +\frac{\pi }{2}\right)+\mathrm{si}\left(\Psi -\frac{\pi }{2}\right)\right]$$
  where the phase distribution is given by
  (9) $$\Psi =\pi \left(\frac{x_{a}}{\lambda_{c}}\right)\sin\left(\phi_{s}\right)$$

The angle measured from radar antenna boresight axis is $\phi_{s}$, $x_{a}$ is the width of the aperture in the azimuth angular direction, and $\mathrm{si}\left(x\right)=\frac{\sin(x)}{x}$. The maximum gain $G_{0}$ of an antenna is proportional to its physical size $A=x_{a}\cdot y_{a}$, which is taken into account by its effective aperture $A_{e}=A\cdot \rho_{a}$, where $\rho_{a}$ is the antenna efficiency. Thus, the gain is calculated as [46] (p. 71).

(10) $$G_{0}=\frac{4\pi A_{e}}{\lambda_{c}^{2}}$$

The gain of an antenna with a rectangular aperture in both angular directions $\vartheta_{s}$ and $\phi_{s}$ can then be calculated for the cosine aperture distribution function as follows [53]:

(11) $$G\left(\vartheta_{s},\phi_{s}\right)=\frac{4\pi \rho_{a}x_{a}y_{a}}{\lambda_{c}^{2}}|E\left(y_{a},\vartheta_{s},\lambda_{c}\right)\cdot E\left(x_{a},\phi_{s},\lambda_{c}\right){|}^{2}$$

Finally, the amplitude weighting function of the antenna characteristic is incorporated via the radar equation given in (5) and (6) by calculating the antenna gain for the receiver $G_{Rx}$ and transmitter $G_{Tx}$ antennas. However, in the case of monostatic radar geometry, they can be assumed to be equal.

**Figure 4:**
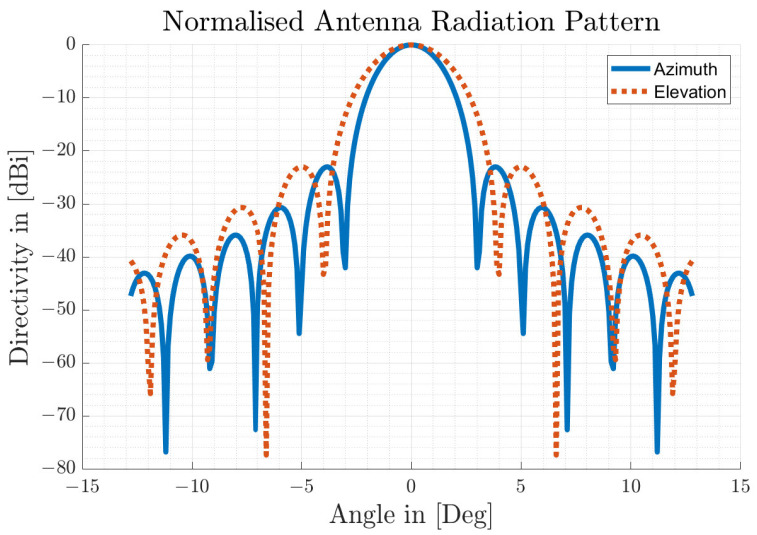
Synthetic antenna radiation pattern.

**Figure 5:**
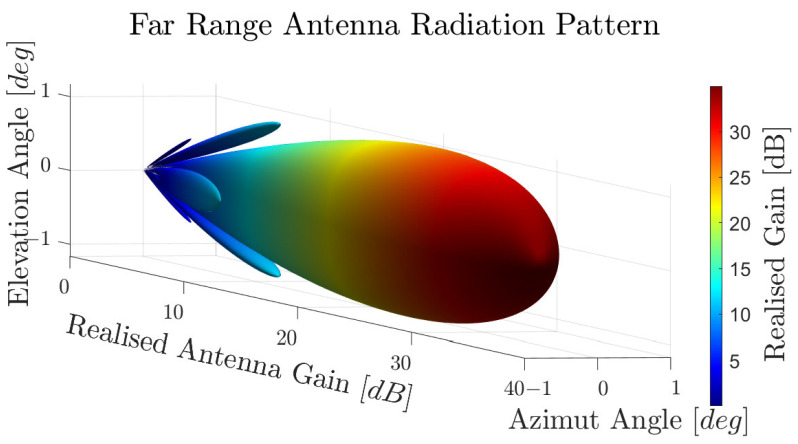
Synthetic antenna pattern in 3D plot.

Figure 4 shows the achieved synthetic antenna radiation pattern in the azimuth direction (blue/solid) and in the elevation direction (red/dashed) on a logarithmic scale, while Figure 5 shows the 3D antenna gain pattern in a 3D plot.

- Propagation factor

The link between the environmental components and the radar sensor is the transmission path through which the RF waves propagate. The characteristics of the propagation channel can vary from direct line-of-sight to partial or complete obstruction by other road users under varying weather conditions. Therefore, propagation channel modelling is usually conducted on a statistical basis [46] (p. 64). In general, the random behaviour of the propagation channel is the combination of the impacts of a number of physical effects described by the propagation factor *F* [52] (p. 118). For the modelling, the propagation channel was empirically analysed according to the EuroNCAP Global Vehicle Target validation procedure [43] (Appendix A3, Figure A8, p. 21) using the real radar sensor.

The expected fluctuations in the received signal strength should vary by three-to-four orders of magnitude, even if the radar geometry changes by only a small fraction of the wavelength. In contrast to this expectation, the experiment revealed a pattern similar to the so-called multi-path fading.

In order to reproduce the measured RCS pattern for each EC considering the monostatic radar geometry, two basic propagation phenomena, reflection and scattering, were considered with respect to geometry, position, and range parameters according to the theoretical background of multi-path EM wave propagation [52] (p. 142). Consequently, for each range bin in each receiver channel occupied by the bounding boxes of the ECs, the two-ray ground reflection model was applied. Furthermore, to characterise the propagation channel, the local average signal power measured over a distance of several multiples of the wavelength can be calculated [46] (p. 70). As the wavelength of mm-Wave radar sensors is in the range of a few millimetres, the model presented averages of the simulated received signal strength over the physical length of the EC rather than over several multiples of the wavelength. This is given by the number of occupied range bins in each receiver channel. The two-way propagation factor for a monostatic radar geometry is given by [52] (p. 143)

(12) $$F^{4}={\left|1+2\cdot \Gamma \cdot \cos\left(k_{\lambda }\Delta R\right)+{\left(\Gamma \cdot \cos\left(k_{\lambda }\Delta R\right)\right)}^{2}\right|}^{2}\;,$$

where 1 is the magnitude of the E-field in the direct path, Γ the complex Fresnel reflection coefficient of the surface, $k_{\lambda }$ is the wavenumber and ΔR is the difference in range between the direct path and the reflected path. The reflection coefficient Γ is a function of the boundary admittance and the angle of incidence, it depends on the carrier frequency of transmitted signal, expressed to vertical and horizontal E-field polarisation to the plane of incidence [46] (p. 79). The Fresnel reflection coefficient Γ we incorporated into the simulation is given according to [52] (p. 148),

(13) $$\Gamma =\Gamma_{0}\cdot \left(\rho_{s}+\rho_{d}\right)\;,$$

where $\Gamma_{0}$ is a smooth earth reflection coefficient, $\rho_{s}$ represents the specular while $\rho_{d}$ represents the diffuse roughness factor. A detailed description of theoretical background and derivation of equations can be found in [52] (Chapter 4, p. 117).

After calculating the signal amplitude in each resolution bins for all ECs, the joint RA map is calculated by summing the individual amplitude values.

#### 3.3.4. Signal Processing

Detection is an automatic decision on the preprocessed signal, comparing received signal strength and spectral peaks against a threshold to determine if the target is in the sensor’s FOV. This process is carried out under the influence of external noise, internal thermal noise of the receiver, interference, and noise jamming from other traffic participants. Due to the fact that noise levels can vary rapidly, an adaptive threshold setting automatically adjusts to the current noise level, maintaining a pre-set false alarm rate [52] (Chapter 15, p. 547). Our modelling approach preprocesses the data by generating a denser set of spatial frequencies, exploiting the negative side-effects of the *FFT interpolation with zero padding*. We choose zero-padding instead of quadratic interpolation [52] (p. 654) because zero padding has the effect of introducing spurious frequency peaks that may not be present in the original input data [49] (p. 75), increasing the uncertainty of the received signal.

In the subsequent processing step, the *CA-CFAR Detection Process* was implemented to reduce the number of unwanted detections. Based on the characteristics of different CFAR algorithms given in [52] (p. 532) and [54], we implemented the 1D Cell Averaging (CA) and 1D Ordered Statistic (OS) CFAR models for radar target extraction applied to the RA map. Although the 1D CA-CFAR performed well in distance measurement and the 1D OS-CFAR performed well in azimuth detection as reported in the literature, the latter was computationally too expensive. This led to the only implementation of a modified 2D FFT-based realisation of a well-known CA-CFAR algorithm introduced in [54]. Finally, the *association of relative velocity* was defined and implemented. In accordance with the environment component classes defined in Section 3.3.2, four different velocity signals can be distinguished to represent the velocity signals measured by the real sensor. Relative velocity for static and dynamic ECs with respect to the azimuth angle, for semi-dynamic ECs with μDoppler superposition, and for dynamic-plus ECs according to our observations in the real radar measurement.

#### 3.3.5. Error Model

The error model shown in Figure 2 includes the generic back-scattering process to incorporate the influence of the number of pulses received during one CPI in combination with the stochastic nature of EM wave propagation. This process can be described by a statistical superposition of a finite number of uniformly distributed, isotropic, uncorrelated scatterers. The simulation uses a Poisson random process to define the number of contributing scatterers at each simulation step, representing the sum of back-scattered pulses expected from a non-fluctuating target. This simplification can be made by assuming a large number of isotropic scatterers with similar reflection coefficients, since in this case the superposition of the phase term is close to zero [49] (p. 81). In active components including the antenna, the thermal noise is always present. Accordingly, the back-scattered signal is always a combination of noise and the target signal. The power spectral density of the thermal noise is constant, and the noise power is uniformly distributed over the range of frequencies in which the radar operates defined by the receiver bandwidth *B*. Since in our sensor model we consider the coherent integration of a received pulse, the signal-to-noise ratio (SNR) for a CPI consisting of $N_{CPI_{RX}}$ pulses can be calculated as the SNR of one pulse times the number of pulses $N_{CPI_{RX}}$ [49] (p. 77). In the simulation, the thermal noise is considered as an additive complex white Gaussian Noise. In reality, due to the acceleration or jerk of the target vehicle, the Doppler frequency spectrum of a moving target is spread over several frequency bins [49] (p. 77). Since a linear phase change is assumed, there is an error in the radial velocity estimate due to Doppler frequency migration (DFM) [52] (p. 841).

### 3.4. Implementation in Matlab

**Figure 6:**
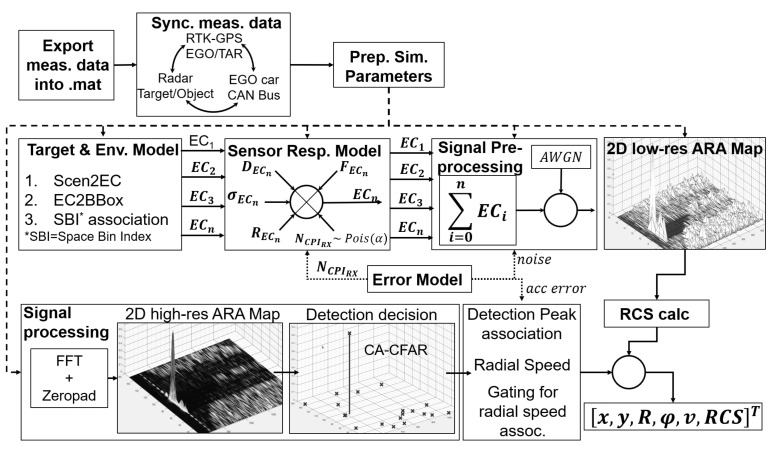
Implementation of the model.

In this paper, we use Matlab/Simulink as a modelling, simulation, and implementation platform. Figure 6 shows the schematic representation of the implementation of the radar sensor model according to the higher-level model described in detail in the previous chapter.

The implementation starts with the export of measured data and synchronising the different datasets from Real Time Kinematics/Global Positioning System (RTK-GPS) measurements (ego and target vehicle) to the CAN bus signals of the ego vehicle and the output of the radar sensor represented by target (radar point clouds) and object lists. From this, the simulation parameters for the replay of the experimental tests in the simulation is prepared. The remainder of Figure 6 follows the methodology of the sensor model approach divided into Target and Environment Mode, Section 3.3.2, the Sensor Response Model, Section 3.3.3, the Signal Processing module, Section 3.3.4, as well as the Error Model, Section 3.3.5, that affects both signal processing and sensor response model modules. The block 2D low-res ARA (amplitude range azimuth) map visualises the results of the low-resolution detection from which the RCS is calculated and associated with the results from the signal processing module.

The final result is the simulated output vector ${\left[x,y,R,\varphi ,v,RCS\right]}^{-T}$ that is compared to the corresponding quantities from measurement in the next section, where x,y,R,φ are coordinates of the radar point cloud in the (Cartesian and polar) sensor coordinate system, *v* the radial velocity, and RCS the radar cross-section.

## 4. Results

In this chapter, we present the simulation results of the radar sensor model described in Section 3 compared to its real sensor counterpart by referencing both datasets to the ground truth (GT) as measured with the RTK-GPS reference system. Since the radar sensor model was implemented in MATLAB/SIMULINK, the virtual replaying of the scenario is also performed here based on the vehicle position and dynamic information provided by the GT system. In addition to simulation fidelity, model verification should also take into account simulation efficiency in terms of real-time capability. An HP Z-Book workstation with an Intel(R) Core(TM) i7-6700HQ CPU @ 2.60 GHz and 16.0 GB of installed RAM was used for the development and simulation of the results presented. The operating system used was a 64-bit version of Windows 10. To illustrate the simulation’s efficiency, the “Range-Test-Target-Leaving” manoeuvre is used. Here, data acquisition begins when the initial conditions are met. The target vehicle then accelerates, leaves the ego vehicle, and ends when it leaves the sensor’s field of view. The duration of the manoeuvre is 160.8 s, during which 2396 radar measurements have been collected. The simulation performance result is summarised in Table 1. The numbers in the first column refer to the modules as shown in Figure 2 in the following way: ① represents the Targets and Environment module, ② the Sensor Response module, and ③ the Signal-Processing module.

As can be seen, the requirement for simulation efficiency in sensor modelling was met, as neither increased computing power nor special code optimisation was required to run the simulation faster than real time for all three in Section 1. For more details about sensor classification, please refer to [2].

In contrast to the real radar sensor, which consists of two sensor parts, one for the near-range and the other for the far-range measurements [45], the far-range characteristics of the RUS are reproduced and calibrated. Since the detection output of the real radar sensor is divided into a far and a near detection range, the evaluation of the synthetic radar data is carried out similarly as in [42]. The near-range detection sector is defined from 0 to 60 m, the far-range detection sector is from 60 to 202.5 m.

### 4.1. Evaluation of Modelling Approach

**Figure 7:**
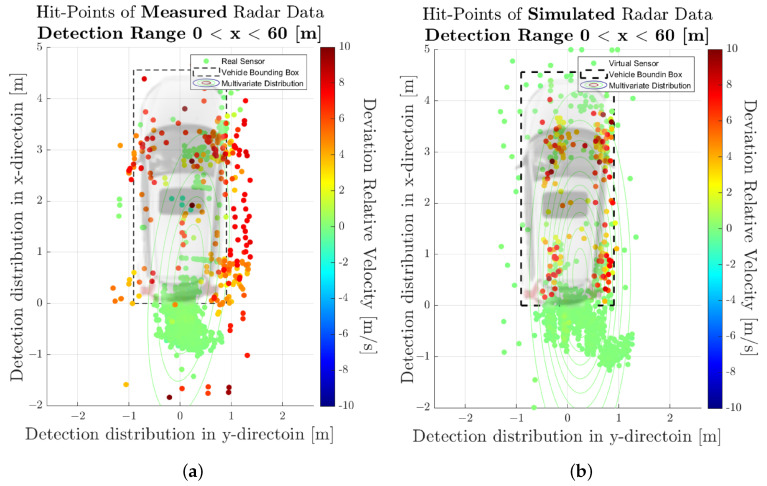
Visualisation of the measured and simulated radar point cloud data, accumulated over the entire measurement time, in the near-range detection sector. (**a**) Scatterplot of detections for the real sensor. (**b**) Scatterplot of detections for the sensor model.

**Figure 8:**
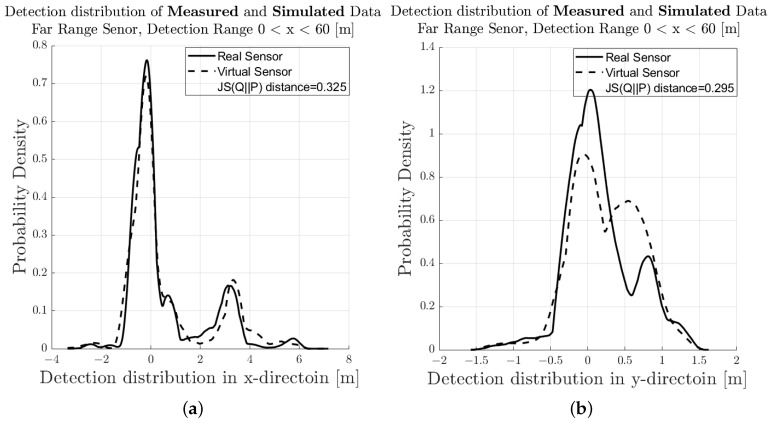
Visual comparison and evaluation of the PDF of the deviation of the measured and simulated radar point clouds with respect to the reference point in the near-range detection sector. (**a**) PDF of the deviation in the *x*-direction from $P_{ref}\left(x,y\right)$ of the real sensor and sensor model. (**b**) PDF of the deviation in the *y*-direction from $P_{ref}\left(x,y\right)$ of the real sensor and sensor model.

**Figure 9:**
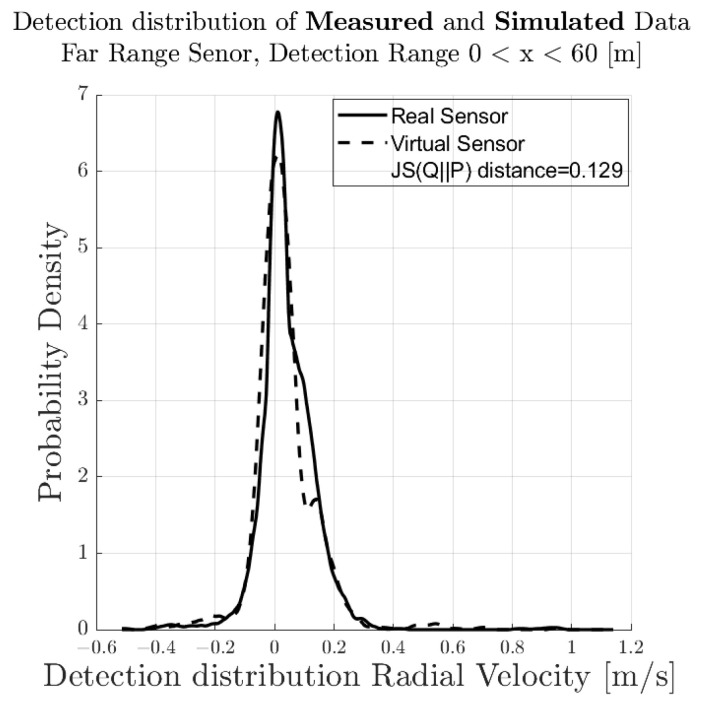
Visual comparison and evaluation of the probability distribution function (PDF) of the deviation of the measured and simulated radar point clouds with respect to the reference point $P_{ref}\left(x,y\right)$ in the near-range detection sector: PDF of the radial velocity deviation from the reference velocity.

Figure 7 shows the spatial distribution of the measured (a) and synthetic (b) radar point cloud for a near-range detection sector, on a 2D plane in the sensor coordinate system related to the dynamic target vehicle. The colour code of each radar detection point (Hit-point) indicates its velocity deviation with respect to the measured radial velocity provided by the GT reference measurement system. The dashed line shows the boundary line of the target vehicle, and the contour lines in this diagram represent the multivariate distribution of the radar point cloud. These figures make it easy to understand that, in addition to the evident radar detections at the rear of the vehicle, there are also detections that can be attributed to the installation position of the wheels. Figure 8 shows the PDFs of the divergence from the reference point $P_{ref}\left(x,y\right)$ in the *x*- and *y*-direction of the measured and simulated radar point cloud. The reference point $P_{ref}\left(x,y\right)$ is provided by the GT reference system, for more details refer to [42]. The probability distribution can not only be used to qualitatively assess the distribution of the reflections, but also serves as a basis for calculating the Jensen–Shannon divergence. Figure 8a shows the divergence in the longitudinal direction and Figure 8b the divergence in the transverse direction. Figure 9 shows a PDF of the divergence of the radial velocity of each radar detection point compared to the GT relative velocity of the target vehicle.

To quantitatively evaluate the performance of the sensor model in terms of its accuracy or fidelity, the Jensen–Shannon divergence (JSD) can be calculated, which is the core part of the DGT-SMV method introduced in [42]. The Jensen–Shannon divergence (JSD) measures the distance between the discrete distribution of the accuracy reference (real sensor data) and the synthetic radar data by comparing their shape. It is a real mathematical metric that always returns the value of DistJS(P||Q) as a scalar number in the closed interval between 0 and 1. If the result of DistJS(P||Q)= 0, the two distributions P and Q are equal; otherwise, if the result of DistJS(P||Q)= 1, they differ as much as possible.

### 4.2. Performance Assessment—Radar Cross-Section

This section shows the simulation result of the synthetically generated RCS output signal. The evaluation of the simulated RCS output as well as the measurement and measurement evaluation of the real sensor output already mentioned in Section 3.3.3 was based on the method presented in [43] and is briefly summarised here. The RCS value measured over the radial distance of a target vehicle is subject to fluctuation in signal amplitude due to the constructive and destructive interference caused by the multipath propagation of EM waves. This fluctuation can lead to very low RCS values at certain distances compared to the typical or expected RCS value. To incorporate this phenomenon into the Euro NCAP target validation process, the assessment is performed by setting an upper and lower limit based on the curve fitting function applied to the measured RCS data.

**Figure 10:**
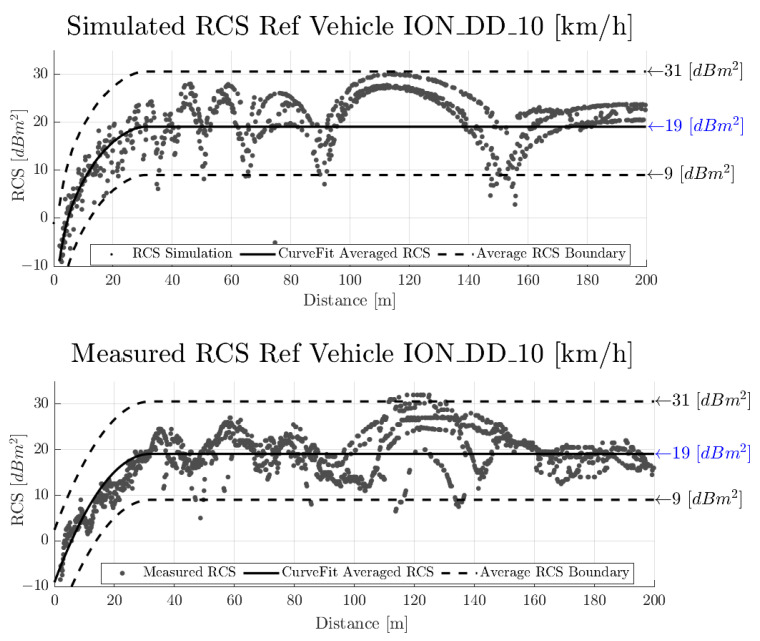
Evaluation of the synthetically generated (**top**) and the measured real radar point cloud (**bottom**) in terms of the radar cross-section signal (RCS), following the procedure described in [43].

The lower plot in Figure 10 shows the results of the RCS measurement, the upper plot shows the synthetic RCS data simulated with the radar sensor model presented in this paper. For evaluation purposes, the result of curve fitting applied to both the measured and simulated RCS data is also shown with the solid line, while the dashed lines represent the lower and upper limits of acceptable fluctuation.

## 5. Discussion and Conclusion

### 5.1. Comparison of Measurement and Simulation

The visual inspection of the accumulated radar point clouds as presented in Figure 7 shows a qualitatively good comparability of the real and simulated radar, both for the position of reflections in *x* and *y* and radial velocity. The attribution of PDFs to these data reveals the nice correlation of the simulation in the longitudinal direction as well as for the radial velocity, as seen in Figure 8 and Figure 9; for the lateral direction, the comparability is still acceptable. This visual impression is confirmed by calculating a quantitative performance number using the JSD metric.

For the RCS values, the visual inspection of Figure 10 reveals good correlations between measurement and simulation. It is worth noting that the accuracy of the GT referencing system does not influence the results, since the comparison is drawn directly between the experiment and the simulation.

In order to objectify these impressions, we compare the findings against results from a commercial radar sensor model in the next section.

### 5.2. Comparison to Commercial Applications

Results with a commercial radar sensor model (IPG CarMaker RSI radar sensor model V8.1.1, [55]) were drawn in previous works, [42]. Since exact the same methodology to benchmark the sensor model was applied, the results are directly comparable. The default values for the model parameters of the commercial sensor were used, since a sensitivity study revealed that tuning the parameters did not change the results significantly.

**Figure 11:**
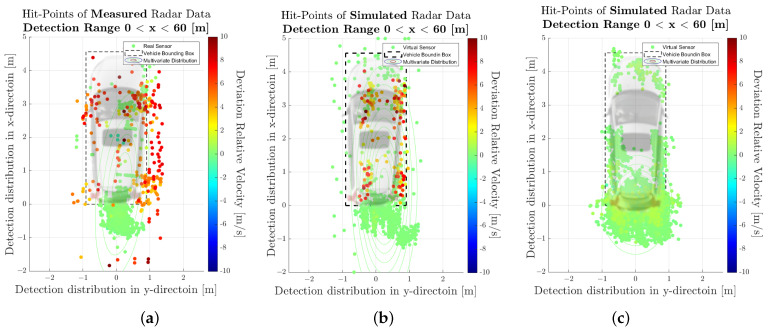
Visualisation of the measured and simulated radar point cloud data, cumulated over the entire measurement time, in the near-range detection sector. (**a**) Scatterplot of detections for the real sensor. (**b**) Scatterplot of detections for the sensor model presented in this paper. (**c**) Scatterplot of detections for the sensor model of commercial application.

**Figure 12:**
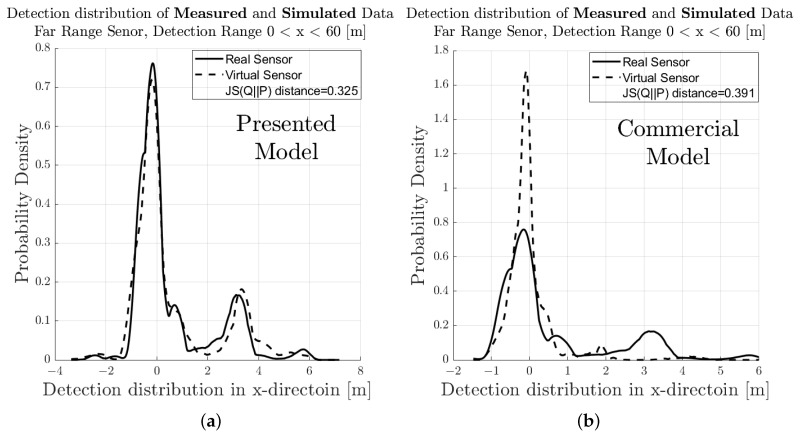
Visual comparison and evaluation of the probability distribution function (PDF) of the deviation of the measured and simulated radar point clouds with respect to the reference point in the near-range detection sector. (**a**) PDF of the deviation in the *x*-direction from $P_{ref}\left(x,y\right)$ of the real sensor and sensor model presented in this paper. (**b**) PDF of the deviation in the *x*-direction from $P_{ref}\left(x,y\right)$ of the real sensor and sensor model for commercial applications.

**Figure 13:**
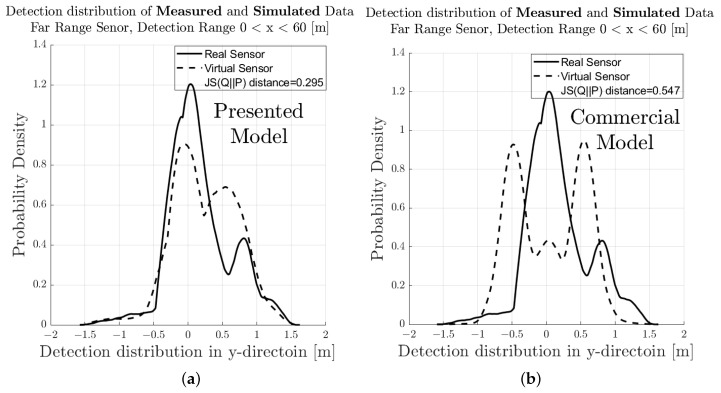
Visual comparison and evaluation of the probability distribution function (PDF) of the deviation of the measured and simulated radar point clouds with respect to the reference point in the near-range detection sector. (**a**) PDF of the deviation in the *y*-direction from $P_{ref}\left(x,y\right)$ of the real sensor and sensor model presented in this paper. (**b**) PDF of the deviation in the *y*-direction from $P_{ref}\left(x,y\right)$ of the real sensor and sensor model for commercial applications.

**Figure 14:**
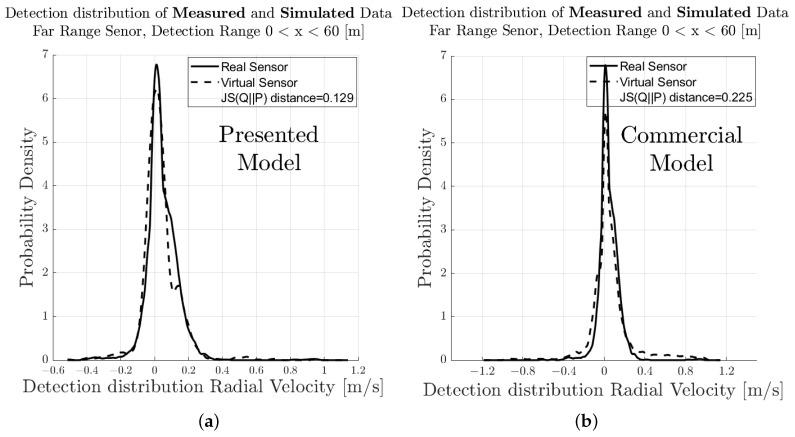
Visual comparison and evaluation of the probability distribution function (PDF) of the deviation of the measured and simulated radar point clouds with respect to the reference point $P_{ref}\left(x,y\right)$ in the near-range detection sector. (**a**) PDF of the radial velocity deviation from the reference velocity of the real sensor and sensor model presented in this paper. (**b**) PDF of the radial velocity deviation from the reference velocity of the real sensor and sensor model for commercial applications.

In Figure 11, the related results for the radar point clouds are observable. It can be clearly seen that the radar point clouds of the commercial sensor differs strongly from the measurements and are concentrated around the rear of the vehicle. The same holds for the comparison of the PDFs, as seen in Figure 12, Figure 13 and Figure 14 which show a rather divergent result for the commercial sensor model. The visual impression is also supported by the JSD metrics. Here, the presented model outperforms the commercial one in almost all aspects but the relative distance in *x* in the far range. This can be explained that some reflection points are observable in the presented model which did not appear in the measurement. Results for the RCS cannot be compared since the commercial model does not deliver those, which is a major drawback for subsequent perception algorithms that lead to object lists. Hence, perception algorithms cannot be applied for the commercial model in a realistic manner.

### 5.3. Outlook

In addition to fine-tuning of the model, e.g., to improve the performance in the far range, future research will address the implementation of the model in commercial software for virtual ADAS/AD validation and verification. Here, the interface to objects as present in the virtual test drives need to be defined. For radar sensors, not only the size of the objects needs to be addressed but also the reflectivity of the surface material. Another future aspect is the application for X-in-the-loop applications; the design of the model is thereof well suited, for example, to calculate radar point clouds to be reproduced by over-the-air radar target simulators. Finally, the influence of weather conditions will be investigated by repeating test drives of different complexity in well-defined adverse weather conditions which requires, for example, outdoor rain simulation. Repeating defined test scenarios in on-road tests will deliver more information to include stochastic results in the evaluation method.

## Figures and Tables

**Table 1:** Result of simulation performance test.

Modules	Classifies as	Execution Time (s)	Speed of Execution
①	OM	29.35	∼5.4 × RT (Real-Time)
① + ②	TM	40.12	∼4.0 × RT
① + ② + ③	FM	114.67	∼1.4 × RT

## Data Availability

Not applicable.

## Abbreviations

The following abbreviations are used in this manuscript:

ADAS	Advanced Driver Assistance Systems
AD	Automated Driving
ADF	Automated Driving Functions
ARA	Amplitude Range Azimuth
ARS-308	Continental Automotive Radar of Series 308
CAN	Controller Area Network
CA	Cell Averaging
CFAR	Continuous False Alarm Rate
DC	Detection Classes
DGT-SMV	Dynamic Ground Truth—Sensor Model Validation
DFM	Doppler Frequency Migration
DL	Deep Learning
CPI	Coherent Process Interval
EuroNCAP	European New Car Assessment Program
EC	Environment Components
FFT	Fast Fourier Transformation
FM	Functional Model
FMCW	Frequency Modulated Continuous Wave (Radar)
FOV	Field of View
GT	Ground Truth
HAR	Human Activity Recognition
HW/SW	Hardware/Software
IM	Individual Model
JSD	Jensen–Shannon Divergence
LTI	Linear Time Invariant System
LOS	Line Of Sight
ML	Machine Learning
RA	Range Azimuth
RCS	Radar Cross-Section
RF	Radio Frequency
RNN	Recurrent Neural Network
RT	Ray Tracing
RTK–GPS	Real-Time Kinematics–Global Positioning System
RUS	Radar Under Simulation
Rx/Tx	Receiver/Transmitter
SAE	Society of Automotive Engineers
SB	Space Bin
SBI	Space Bin Indicator
SNR	Signal-to-Noise Ratio
TM	Technical Model
OEM	Original Equipment Manufacturer, i.e., Vehicles Manufacturer
OM	Operational Model
OS-CFAR	Ordered Statistic Continuous False Alarm Rate
OTA	Over-the-Air (Radar Target Simulators)
PDF	Probability Density Function
V&V	Validation (of intended use) and Verification (of requirements)

## References

[1] Kalra N., Paddock S.M. (2016). Driving to Safety: How Many Miles of Driving Would It Take to Demonstrate Autonomous Vehicle Reliability?. Transp. Res. Part A Policy Pract..

[2] Magosi Z.F., Li H., Rosenberger P., Wan L., Eichberger A. (2022). A Survey on Modelling of Automotive Radar Sensors for Virtual Test and Validation of Automated Driving. Sensors.

[3] Hartstern M., Rack V., Kaboli M., Stork W. Simulation-based Evaluation of Automotive Sensor Setups for Environmental Perception in Early Development Stages. Proceedings of the 2020 IEEE Intelligent Vehicles Symposium (IV).

[4] Hanke T., Schaermann A., Matthias G., Konstantin W., Hirsenkorn N., Rauch A., Schneider S.A. Generation and validation of virtual point cloud data for automated driving systems. Proceedings of the 20th International Conference on Intelligent Transportation Systems.

[5] Linnhoff C., Rosenberger P., Holder M.F., Cianciaruso N., Winner H., Bertram T. (2021). Highly Parameterizable and Generic Perception Sensor Model Architecture. Automatisiertes Fahren 2020.

[6] Rosique F., Navarro P.J., Fernández C., Padilla A. (2019). A Systematic Review of Perception System and Simulators for Autonomous Vehicles Research. Sensors.

[7] Muckenhuber S., Museljic E., Stettinger G. (2021). Performance evaluation of a state-of-the-art automotive radar and corresponding modelling approaches based on a large labeled dataset. J. Intell. Transp. Syst..

[8] Hanke T., Hirsenkorn N., Dehlink B., Rauch A., Rasshofer R., Biebl E. Generic architecture for simulation of ADAS sensors. Proceedings of the 2015 16th International Radar Symposium (IRS).

[9] Jun Z., Kai Y., Xuecai D., Zhangu W., Huainan Z., Chunguang D. (2021). New modeling method of millimeter-wave radar considering target radar echo intensity. Proc. Inst. Mech. Eng. Part D J. Automob. Eng..

[10] Bernsteiner S., Magosi Z., Lindvai-Soos D., Eichberger A. (2015). Radar Sensor Model for the Virtual Development Process. ATZelektronik Worldw..

[11] Thieling J., Frese S., RoBmann J. (2021). Scalable and Physical Radar Sensor Simulation for Interacting Digital Twins. IEEE Sens. J..

[12] Schuesler C., Hoffmann M., Braunig J., Ullmann I., Ebelt R., Vossiek M. (2021). A Realistic Radar Ray Tracing Simulator for Large MIMO-Arrays in Automotive Environments. IEEE J. Microwaves.

[13] Dudek M., Wahl R., Kissinger D., Weigel R., Fischer G. Millimeter wave FMCW radar system simulations including a 3D ray tracing channel simulator. Proceedings of the 2010 Asia-Pacific Microwave Conference.

[14] Chipengo U. (2018). Full Physics Simulation Study of Guardrail Radar-Returns for 77 GHz Automotive Radar Systems. IEEE Access.

[15] Zhu L., He D., Ai B., Zhong Z., Zhu F., Wang Z. Measurement and Ray-Tracing Simulation for Millimeter-Wave Automotive Radar. Proceedings of the 2021 IEEE 4th International Conference on Electronic Information and Communication Technology (ICEICT).

[16] Maier F.M., Makkapati V.P., Horn M. (2018). Environment perception simulation for radar stimulation in automated driving function testing. E I Elektrotechnik Und Informationstechnik.

[17] Maier M., Makkapati V.P., Horn M. Adapting Phong into a Simulation for Stimulation of Automotive Radar Sensors. Proceedings of the 2018 IEEE MTT-S International Conference on Microwaves for Intelligent Mobility (ICMIM).

[18] Holder M.F., Makkapati V.P., Rosenberger P., D’hondt T., Slavik Z., Maier F.M., Schreiber H., Magosi Z., Winner H., Bringmann O. Measurements revealing Challenges in Radar Sensor Modeling for Virtual Validation of Autonomous Driving. Proceedings of the 2018 21st International Conference on Intelligent Transportation Systems (ITSC).

[19] Hirsenkorn N., Subkowski P., Hanke T., Schaermann A., Rauch A., Rasshofer R., Biebl E. A ray launching approach for modeling an FMCW radar system. Proceedings of the 18th International Radar Symposium IRS 2017.

[20] Martin M.Y., Winberg S.L., Gaffar M.Y.A., Macleod D. (2022). The Design and Implementation of a Ray-tracing Algorithm for Signal-level Pulsed Radar Simulation Using the NVIDIA® OptiXTM Engine. J. Commun..

[21] Holder M., Linnhoff C., Rosenberger P., Winner H. The Fourier Tracing Approach for Modeling Automotive Radar Sensors. In Proceedings of the 20th International Radar Symposium (IRS).

[22] Schubert R., Mattern N., Bours R. (2014). Simulation of Sensor Models for the Evaluation of Advanced Driver Assistance Systems. ATZelektronik Worldw..

[23] Li H., Kanuric T., Eichberger A. (2022). Automotive Radar Modeling for Virtual Simulation Based on Mixture Density Network. IEEE Sens. J..

[24] Owaki T., Machida T. Hybrid Physics-Based and Data-Driven Approach to Estimate the Radar Cross-Section of Vehicles. Proceedings of the 2019 IEEE Intelligent Transportation Systems Conference (ITSC).

[25] Hirsenkorn N., Hanke T., Rauch A., Dehlink B., Rasshofer R., Biebl E. A non-parametric approach for modeling sensor behavior. Proceedings of the 2015 16th International Radar Symposium (IRS).

[26] Hirsenkorn N., Hanke T., Rauch A., Dehlink B., Rasshofer R., Biebl E. (2016). Virtual sensor models for real-time applications. Adv. Radio Sci..

[27] Choi W.Y., Yang J.H., Chung C.C. (2021). Data-Driven Object Vehicle Estimation by Radar Accuracy Modeling with Weighted Interpolation. Sensors.

[28] Moujahid A., Tantaoui M.E., Hina M.D., Soukane A., Ortalda A., ElKhadimi A., Ramdane-Cherif A. Machine learning techniques in ADAS: A review. Proceedings of the 2018 International Conference on Advances in Computing and Communication Engineering (ICACCE).

[29] Sligar A.P. (2020). Machine Learning-Based Radar Perception for Autonomous Vehicles Using Full Physics Simulation. IEEE Access.

[30] Rathi A., Deb D., Sarath Babu N., Mamgain R., Zhang Y.D., Mandal J.K., So-In C., Thakur N.V. (2020). Two-level Classification of Radar Targets Using Machine Learning. Smart Trends in Computing and Communications.

[31] Abeynayake C., Son V., Shovon M., Yokohama H., Bishop S.S., Isaacs J.C. (2019). Machine learning based automatic target recognition algorithm applicable to ground penetrating radar data. Proceedings of the Detection and Sensing of Mines, Explosive Objects, and Obscured Targets XXIV.

[32] Carrera E.V., Lara F., Ortiz M., Tinoco A., León R. Target detection using radar processors based on machine learning. Proceedings of the 2020 IEEE ANDESCON.

[33] Shinde P.P., Shah S. A review of machine learning and deep learning applications. Proceedings of the 2018 Fourth International Conference on Computing Communication Control and Automation (ICCUBEA).

[34] Chen K., Yao L., Zhang D., Wang X., Chang X., Nie F. (2020). A Semisupervised Recurrent Convolutional Attention Model for Human Activity Recognition. IEEE Trans. Neural Netw. Learn. Syst..

[35] Zhang D., Yao L., Chen K., Wang S., Chang X., Liu Y. (2019). Making sense of spatio-temporal preserving representations for EEG-based human intention recognition. IEEE Trans. Cybern..

[36] Arefnezhad S., Hamet J., Eichberger A., Frühwirth M., Ischebeck A., Koglbauer I.V., Moser M., Yousefi A. (2022). Driver drowsiness estimation using EEG signals with a dynamical encoder–decoder modeling framework. Sci. Rep..

[37] Song Y., Wang Y., Li Y. (2019). Radar data simulation using deep generative networks. J. Eng..

[38] Slavik Z., Mishra K.V. Phenomenological Modeling of Millimeter-Wave Automotive Radar. Proceedings of the 2019 URSI Asia-Pacific Radio Science Conference (AP-RASC).

[39] Schuler K., Becker D., Wiesbeck W. (2008). Extraction of Virtual Scattering Centers of Vehicles by Ray-Tracing Simulations. IEEE Trans. Antennas Propag..

[40] Lutz S., Ellenrieder D., Walter T., Weigel R. On fast chirp modulations and compressed sensing for automotive radar applications. Proceedings of the 2014 15th International Radar Symposium (IRS).

[41] Schneider R., Wenger J. (2003). High resolution radar for automobile applications. Advances in Radio Science.

[42] Magosi Z.F., Wellershaus C., Tihanyi V.R., Luley P., Eichberger A. (2022). Evaluation Methodology for Physical Radar Perception Sensor Models Based on On-Road Measurements for the Testing and Validation of Automated Driving. Energies.

[43] EuroNCAP (2018). Technical Bulletin TB025 Global Vehicle Target Specification v1.0. https://cdn.euroncap.com/media/39159/tb-025-global-vehicle-target-specification-for-euro-ncap-v10.pdf.

[44] Li Y., Du L., Liu H. (2013). Hierarchical Classification of Moving Vehicles Based on Empirical Mode Decomposition of Micro-Doppler Signatures. IEEE Trans. Geosci. Remote Sens..

[45] Continental Engineering Services GmbH (2012). ARS 308-2C/-21.

[46] Rappaport T.S. (1996). Wireless Communications: Principles and Practice.

[47] Rabe H. (2013). Bildgebende Verfahren zur Steigerung der Ausfallsicherheit Radarbasierter Füllstandsmesssysteme. Ph.D. Thesis.

[48] Fishler E., Haimovich A., Blum R.S., Cimini L.J., Chizhik D., Valenzuela R.A. (2006). Spatial Diversity in Radars—Models and Detection Performance. IEEE Trans. Signal Process..

[49] Koks D. How to Create and Manipulate Radar Range-Doppler Plots: DSTO–TN–1386. https://apps.dtic.mil/sti/pdfs/ADA615308.pdf.

[50] Waldschmidt C., Hasch J., Menzel W. (2021). Automotive Radar—From First Efforts to Future Systems. IEEE J. Microwaves.

[51] Skolnik M.I. (2007). Introduction to Radar Systems.

[52] Richards M.A., Scheer J.A., Holm W.A. (2015). Principles of Modern Radar.

[53] Slocumb B.J., Macumber D.L., Drummond O.E. (2006). Surveillance radar range-bearing centroid processing, part II: Merged measurements. Proceedings SPIE 6236, Signal and Data Processing of Small Targets 2006.

[54] Rohling H. (1983). Radar CFAR Thresholding in Clutter and Multiple Target Situations. IEEE Trans. Aerosp. Electron. Syst..

[55] Herrmann M., Schön H., Bertram T. (2019). Efficient Sensor Development Using Raw Signal Interfaces. Fahrerassistenzsysteme 2018.

[56] Wellershaus C. (2021). Performance Assesment of a Physical Sensor Model for Automated Driving. Master’s Thesis.

